# Spontaneous Coronary Artery Dissection in Fragile X Syndrome: A Novel IVUS-Guided PCI Approach

**DOI:** 10.1016/j.jaccas.2026.110387

**Published:** 2026-09-23

**Authors:** Majid Anwar, Harsh Chawla, Thomas Kaier, Paul Bambrough

**Affiliations:** Department of Cardiology, Royal Papworth Hospital, Cambridge, United Kingdom

**Keywords:** dissection, intravascular ultrasound, percutaneous coronary intervention

## Abstract

**Background:**

Spontaneous coronary artery dissection is an uncommon cause of acute coronary syndromes, with rare reports in fragile X syndrome.

**Case Summary:**

A 44-year-old man with fragile X syndrome presented with ventricular fibrillation arrest and anterior ST-segment elevation. Angiography and intravascular ultrasound (IVUS) revealed extensive left anterior descending artery spontaneous coronary artery dissection with inadvertent false-lumen wiring. A dual-catheter technique was used, with live false-lumen IVUS-guided true-lumen wiring, hematoma fenestration, and ostial stenting, with postprocedural imaging confirming dissection sealing. Computed tomography angiography identified additional silent dissections of the celiac, superior mesenteric, and right femoral arteries.

**Discussion:**

This case suggests systemic vascular fragility in fragile X syndrome and describes a novel IVUS-guided bailout strategy using live false-lumen imaging to facilitate true-lumen wiring and ostial stent positioning.

**Take-Home Messages:**

Acute coronary syndrome in fragile X syndrome should prompt consideration of systemic arteriopathy and multivessel dissection. Live false-lumen IVUS may facilitate true-lumen wiring when conventional techniques fail.

## Visual Summary

**Figure:**
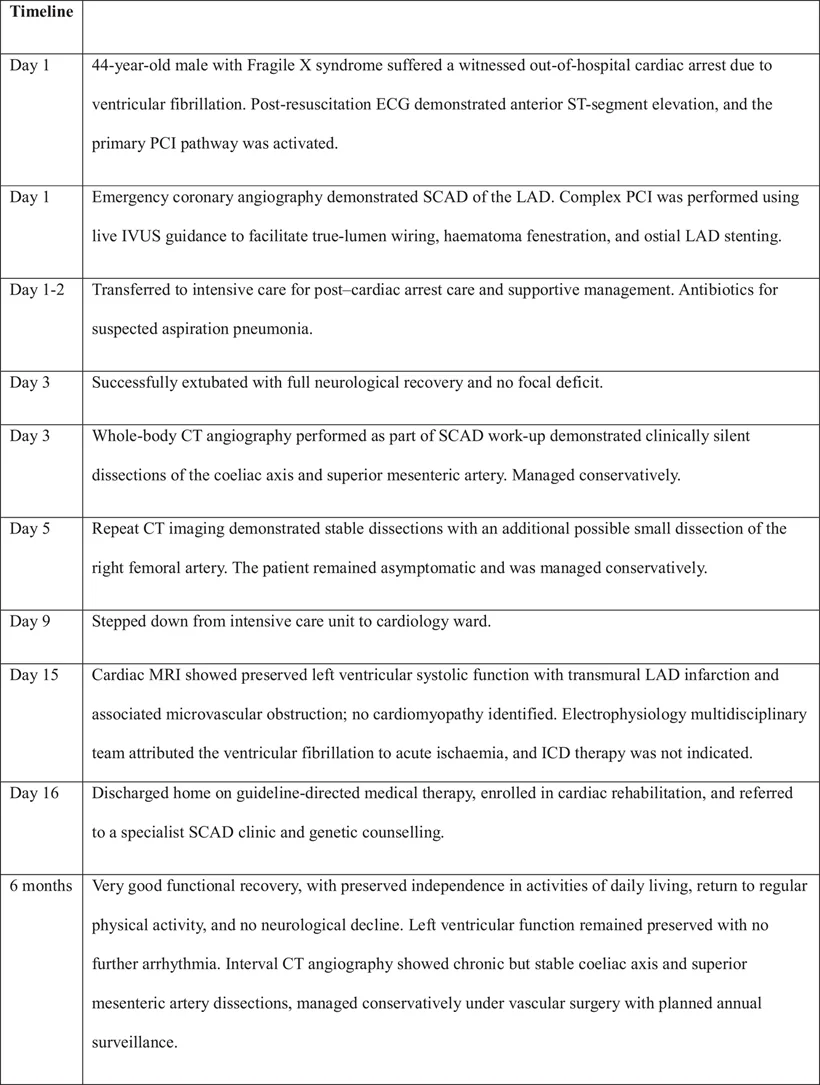

## History of Presentation and Past Medical History

A 44-year-old man with a past medical history of fragile X syndrome, autism, and asthma suffered a witnessed out-of-hospital cardiac arrest because of ventricular fibrillation. Bystander cardiopulmonary resuscitation was started immediately. Paramedics arrived within 5 minutes and administered advanced life support, including adrenaline, amiodarone, intubation, and 7 direct current cardioversion shocks. Return of spontaneous circulation was achieved after 25 minutes.

A postarrest electrocardiogram showed anterior ST-segment elevation, activating the primary percutaneous coronary intervention (PCI) pathway.

The patient arrived in the catheterization laboratory 80 minutes after symptom onset, hemodynamically stable and requiring no inotropic support. Dual antiplatelet therapy was given via nasogastric tube, and echocardiography showed mild left ventricular impairment with a hypokinetic, dyssynchronous septum.

## Investigations and Management

Emergency coronary angiography via the right radial approach showed a nondominant, normal right coronary artery. The left main stem (LMS) was short, with dye hold-up in the left anterior descending (LAD) artery on initial contrast injection; the left circumflex (LCx) appeared normal. The differential included type 4 spontaneous coronary artery dissection (SCAD) vs iatrogenic catheter-induced dissection, but presenting ST-segment elevation and stable hemodynamics after contrast injection favored SCAD.

Despite the consensus favoring conservative management of SCAD whenever possible, PCI was considered necessary in this case because of extensive proximal LAD involvement with impaired flow and a large myocardial territory at risk, in addition to persistent ST-segment elevation and malignant ventricular arrhythmias.^1^

The LAD was wired with a balanced middleweight guidewire through a 6-F XB3 guide catheter without further contrast injection. Intravascular ultrasound (IVUS) confirmed the guidewire had inadvertently entered the false lumen, with a large intramural hematoma compressing the true lumen throughout most of the LAD.

Withdrawing the false-lumen equipment risked repeat dissection propagation with each rewiring attempt. Given the very proximal dissection and risk of side-branch loss in a hemodynamically unstable patient, we instead leveraged the existing IVUS catheter position within the false lumen to guide live antegrade true-lumen wiring, converting a hazardous situation into a controlled revascularization strategy.

Secondary femoral access was obtained for a second 7-F XB3 guide catheter in a ping-pong technique,^2^ maintaining the IVUS catheter in the false lumen via the radial catheter whereas a Sion Blue guidewire advanced from the femoral catheter. Live IVUS imaging from the very proximal LAD false lumen demonstrated the proximal dissection entry point and was used to direct the second guidewire into the true lumen under real-time visualization. The LCx was also protected with a balanced middleweight guidewire.

An undersized 2.5-mm Wolverine cutting balloon fenestrated the intramural hematoma. Given the long dissection length and proximal-to-distal size mismatch, a 2-stage stenting strategy was planned: the proximal LAD and ostium were secured with a 3.5 × 32 mm Megatron drug-eluting stent (DES). Stent placement was guided by live IVUS from the false lumen using differential acoustic reflection patterns of the crimped stent to align the proximal stent edge with the normal proximal vessel. The false-lumen equipment and IVUS were removed before DES deployment. IVUS from the true lumen then identified the distal dissection length beyond the stent outflow, and a further 2.5 × 48 mm Synergy DES was implanted with postdilatation using a 3.5-mm noncompliant balloon.

Final IVUS confirmed good stent expansion and complete sealing of the dissection proximally and distally. Ostial LAD stenting had been achieved with IVUS showing only 2 stent struts protruding back into the LMS bifurcation. IVUS of the LCx into the LMS confirmed normal architecture with no hematoma propagation or impingement from the ostial LAD stent. Final angiography showed TIMI flow grade 3 in all vessels, with complete resolution of ST-segment elevation.

## Outcome and Follow-Up

Following PCI, the patient was transferred to intensive care for postcardiac arrest management, including targeted temperature management. Neurological recovery was favorable, with successful extubation at 48 hours and no residual deficit.

As part of a SCAD workup, whole-body computed tomography angiography revealed clinically silent dissections of the celiac axis and superior mesenteric artery, with repeat imaging at 48 hours showing stable findings and a possible additional small right femoral artery dissection. The patient remained asymptomatic and was managed conservatively, with interval imaging planned at 6 months.

Cardiac magnetic resonance imaging showed normal left ventricular function (ejection fraction 55%), transmural LAD infarction (4/17 segments) with hypokinesis, and no cardiomyopathy. The arrest was attributed to SCAD, and a secondary-prevention implantable cardioverter-defibrillator was not indicated.

The patient was discharged home in stable condition on guideline-directed medical therapy, including dual antiplatelet therapy and beta-blockade, and enrolled in cardiac rehabilitation. Genetic counselling and family testing were arranged given his fragile X syndrome.

At 6-month review, he had made a very good functional recovery, remaining independent, attending day care 4 times weekly, cycling regularly, with no neurological decline. Left ventricular function remained preserved with no further arrhythmia. Interval computed tomography angiography showed chronic but stable celiac axis and superior mesenteric artery dissections (managed conservatively under vascular surgery with planned annual surveillance) and resolution of the right femoral artery dissection (Figure 1).

## Discussion

SCAD is an increasingly recognized cause of acute coronary syndrome in younger patients, arising from an intramural hematoma or intimal tear that compresses the true lumen and can precipitate malignant arrhythmia or death. It has been linked to connective tissue disorders, fibromuscular dysplasia, peripartum states, systemic inflammatory disease, and precipitating stressors.^3^

Fragile X syndrome is primarily known for its neurodevelopmental phenotype, but emerging data suggest connective tissue abnormalities, including vascular fragility, that may predispose to dissection.^4^ It results from fragile X mental retardation 1 gene silencing: expansion of the normal 5-40 cytosine-guanine-guanine repeats to over 200 causes hypermethylation and loss of the fragile X mental retardation protein (FMRP), essential for normal synaptic development.

FMRP deficiency also alters vascular biology, increasing matrix metalloproteinase-9 activity and extracellular matrix degradation,^4^ and disrupting elastin and actin organization, resulting in abnormal elastin matrix architecture within connective tissue.^4^

These underlying molecular mechanisms may explain why patients with fragile X syndrome can present, as in our case, with a systemic arteriopathy characterized by SCAD in addition to clinically silent dissections involving the celiac, superior mesenteric, and femoral arteries, rather than an isolated coronary event. To our knowledge, this represents one of the first reported associations between fragile X syndrome, SCAD, and multivessel arterial dissection.

The combination of FMRP deficiency, increased matrix metalloproteinase activity, and abnormal elastin organization may collectively predispose patients to both spontaneous and iatrogenic arterial dissection. Careful consideration should therefore precede invasive vascular or coronary procedures in this population.

Fragile X syndrome may also predispose to other connective-tissue-related cardiovascular manifestations, including aeropathy and mitral valve prolapse.^5^ Although classically X-linked dominant, rare SCAD cases have been reported in female carriers of the fragile X mutation, and the fragile X premutation (55-200 cytosine-guanine-guanine repeats) has also been associated with connective tissue disorders and SCAD.^6^

Although conservative therapy is preferred whenever feasible in SCAD,^1^ the extensive LAD involvement, persistent ST-segment elevation, and ventricular fibrillation arrest in this case mandated revascularization. The complex procedure combined a ping-pong dual-catheter approach, live IVUS guidance, and cutting-balloon fenestration before stenting and underscored the critical role of intravascular imaging in diagnosis and safe revascularization.^2^^,^^7^

Registry data support cutting-balloon fenestration to decompress the false lumen and restore true-lumen flow when PCI is required.^7^ Intravascular imaging remains essential throughout, confirming fenestration, guidewire position, and appropriate stent sizing to dissection length and vessel diameter.^8^

IVUS-guided subintimal re-entry is well established in complex chronic total occlusion PCI for techniques such as subintimal tracking and re-entry, antegrade dissection and re-entry and reverse controlled antegrade and retrograde tracking.^9^ However, published experience with IVUS-guided wiring strategies in SCAD remains minimal.

In our case, IVUS was initially used to confirm true-lumen wiring, but after it was inadvertently advanced into the false lumen, it was instead leveraged to facilitate true-lumen wiring of the proximal LAD on the second attempt. Live IVUS has previously facilitated ultra-low-contrast PCI and side-branch ostial stenting, but not intentional false-lumen use in SCAD.^10^

In SCAD cases where the IVUS catheter enters the false lumen, it is important to keep the catheter as proximal as possible to avoid propagation of the intramural hematoma and extension of the dissection. Unlike chronic total occlusion PCI, where imaging facilitates false-to-true-lumen re-entry, IVUS in SCAD PCI primarily aims to avoid false-lumen wiring at the outset. Assessment of dissection length should be performed with IVUS imaging obtained from the true lumen, which can be advanced safely with lower risk of further hematoma or dissection propagation.

Distal microcatheter contrast injection is an alternative to confirm true-lumen position but carries similar risk of propagating the dissection if it lies in the false lumen. Careful antegrade wiring therefore remains first-line in SCAD PCI, with false-lumen IVUS reserved as a bailout when this occurs inadvertently.

## Conclusions

This case describes a young male with fragile X syndrome who presented with ventricular fibrillation arrest because of extensive LAD SCAD, complicated by silent extracoronary dissections. Successful revascularization was achieved using a novel IVUS-guided strategy, leveraging false-lumen live imaging to facilitate true-lumen wiring and ostial stenting. The case highlights the importance of intravascular imaging in complex SCAD and the association between fragile X syndrome and systemic vascular fragility.

**Take-Home Messages**

• Patients with fragile X syndrome presenting with acute coronary syndrome warrant a high index of suspicion for systemic arteriopathy and multivessel arterial dissections.
• False-lumen live intravascular ultrasound guidance may serve as a valuable bailout adjunct in spontaneous coronary artery dissection when the intravascular ultrasound catheter is inadvertently advanced into the false lumen and conventional wiring techniques are unsuccessful.

**Visual Summary:** Timeline of the Case

D 1	44-y-old man with fragile X syndrome suffered a witnessed out-of-hospital cardiac arrest because of ventricular fibrillation. Postresuscitation ECG demonstrated anterior ST-segment elevation, and the primary PCI pathway was activated.
D 1	Emergency coronary angiography demonstrated SCAD of the LAD. Complex PCI was performed using live IVUS guidance to facilitate true-lumen wiring, hematoma fenestration, and ostial LAD stenting.
D 1-2	Transferred to intensive care for post–cardiac arrest care and supportive management. Antibiotics for suspected aspiration pneumonia.
D 3	Successfully extubated with full neurological recovery and no focal deficit.
D 3	Whole-body CT angiography performed as part of SCAD workup demonstrated clinically silent dissections of the celiac axis and superior mesenteric artery. Managed conservatively.
D 5	Repeat CT imaging demonstrated stable dissections with an additional possible small dissection of the right femoral artery. The patient remained asymptomatic and was managed conservatively.
D 9	Stepped down from intensive care unit to cardiology ward.
D 15	Cardiac MRI showed preserved left ventricular systolic function with transmural LAD infarction and associated microvascular obstruction; no cardiomyopathy identified. Electrophysiology multidisciplinary team attributed the ventricular fibrillation to acute ischemia, and ICD therapy was not indicated.
D 16	Discharged home on guideline-directed medical therapy, enrolled in cardiac rehabilitation, and referred to a specialist SCAD clinic and genetic counselling.
6 mo	Very good functional recovery, with preserved independence in activities of daily living, return to regular physical activity, and no neurological decline. Left ventricular function remained preserved with no further arrhythmia. Interval CT angiography showed chronic but stable celiac axis and superior mesenteric artery dissections, managed conservatively under vascular surgery with planned annual surveillance.

CT = computed tomography; ECG = electrocardiogram; ICD = implantable cardioverter-defibrillator; IVUS = intravascular ultrasound; LAD = left anterior descending (coronary artery); MRI = magnetic resonance imaging; PCI = percutaneous coronary intervention; SCAD = spontaneous coronary artery dissection.

**Equipment List:** Equipment List Used for Primary PCI Procedure

Access • Ultrasound machine (Mindray) • Merit 6-F radial sheath • Teleflex Arrow 7-F femoral sheath • Terumo TR Band radial compression device • Abbott Vascular Perclose Prostyle Coronary Kit • Diagnostic catheter Cordis 5-F JR4.0 • Guide catheter Cordis 6-F XB 3.0 • Guide catheter Cordis 7-F XB 3.0 • 0.035 J wire • Abbott Vascular Hi-torque balance middleweight guide wire • ASAHI SION blue guide wire • Abbott Vascular Trek semicompliant balloon 2.5 × 12 mm • Boston Scientific Wolverine cutting balloon 2.5 × 10 mm • Boston Scientific Synergy Megatron 3.5 × 32 mm drug eluting stent • Boston Scientific Synergy 2.5 × 48 mm drug eluting stent • Boston Scientific Emerge noncompliant balloon 3.5 × 15 mm Imaging • Ultrasound machine (Mindray) • Boston Scientific AVVIGO multimodality guidance system IVUS and OptiCross 35 IVUS imaging catheter

AVVIGO = Advanced Visualization for Vascular Imaging and Guidance Optimization; IVUS = intravascular ultrasound.

**Figure 1:**
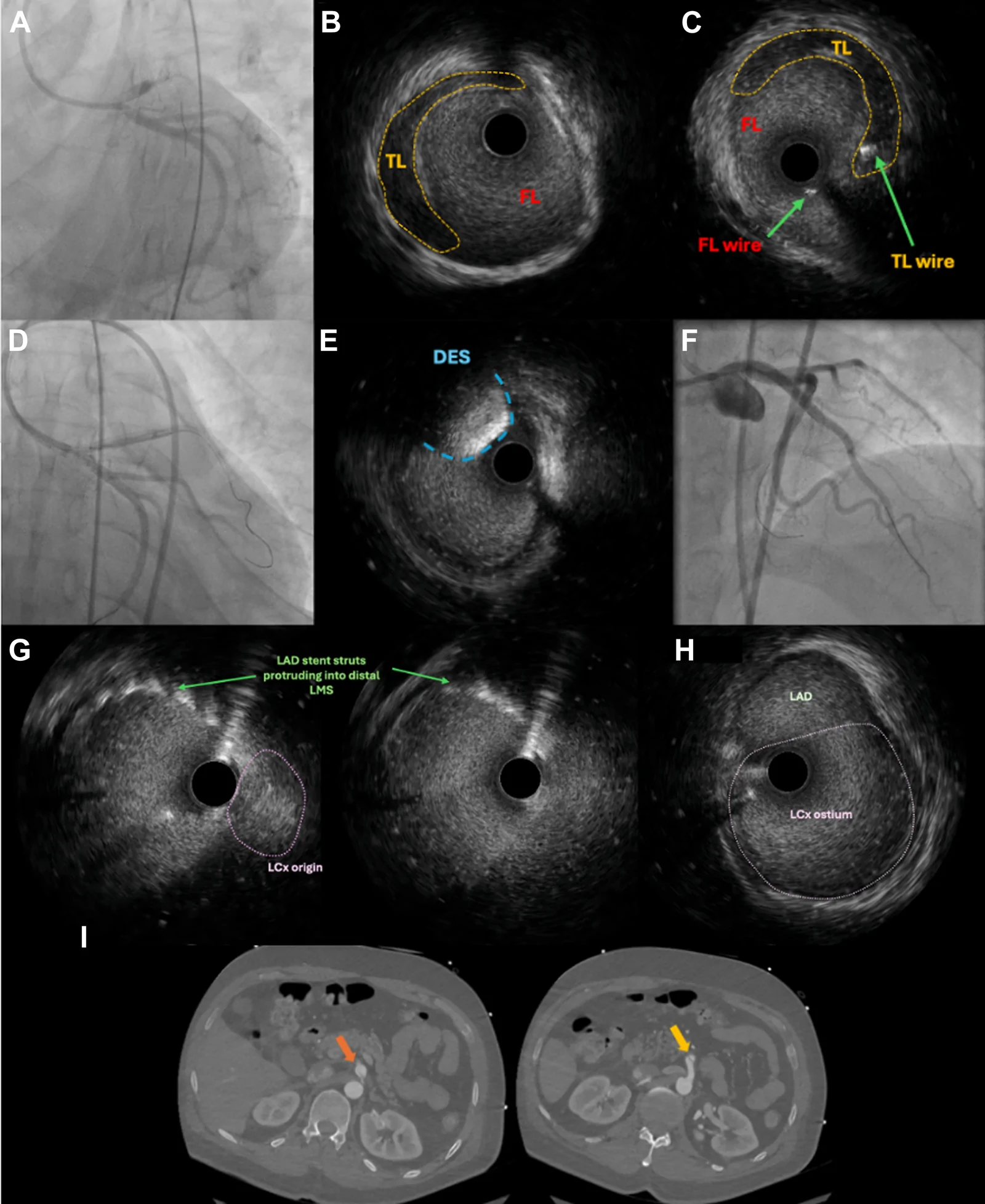
Multimodality Imaging of Spontaneous Coronary Artery Dissection in a Patient With Fragile X Syndrome (A) Coronary angiography showing dye hold-up and staining in the proximal left anterior descending (LAD) artery with reduced flow. (B) Intravascular ultrasound during initial wiring of the LAD confirming guidewire passage into the false lumen (FL) with a large intramural hematoma compressing the true lumen (TL). (C) Live intravascular ultrasound guidance facilitating successful rewiring of the LAD true lumen. (D) Coronary angiography showing positioning of a drug-eluting stent at the LAD ostium, with the intravascular ultrasound catheter visible in the proximal false lumen. (E) Live intravascular ultrasound from the false lumen used to guide precise ostial stent (DES) deployment in the LAD. (F) Final coronary angiogram demonstrating sealing of the LAD dissection with 2 drug-eluting stents and restoration of flow in all vessels in myocardial infarction 3. (G) Final intravascular ultrasound pullback from the LAD into the left main stem (LMS) showing 2 proximal LAD stent struts protruding into the distal LMS without left circumflex (LCx) ostial impingement. (H) Final intravascular ultrasound pullback from the LCx into the LMS confirming a normal, uncompromised LCx ostium with no hematoma propagation or impingement from the ostial LAD stent. (I) Whole-body CT angiography demonstrating extracoronary arterial involvement with dissection of the celiac trunk (orange arrow) and superior mesenteric artery (yellow arrow).

## Funding Support and Author Disclosures

The authors have reported that they have no relationships relevant to the contents of this paper to disclose.
